# Enzymatic transesterification of Jatropha oil

**DOI:** 10.1186/1754-6834-2-1

**Published:** 2009-01-14

**Authors:** Annapurna Kumari, Paramita Mahapatra, Vijay Kumar Garlapati, Rintu Banerjee

**Affiliations:** 1 Agricultural and Food Engineering Department, Indian Institute of Technology, Kharagpur, West Bengal 721302, India

## Abstract

**Background:**

Transesterification of Jatropha oil was carried out in t-butanol solvent using immobilized lipase from *Enterobacter aerogenes*. The presence of t-butanol significantly reduced the negative effects caused by both methanol and glycerol. The effects of various reaction parameters on transesterification of Jatropha oil were studied.

**Results:**

The maximum yield of biodiesel was 94% (of which 68% conversion was achieved with respect to methyl oleate) with an oil:methanol molar ratio of 1:4, 50 U of immobilized lipase/g of oil, and a t-butanol:oil volume ratio of 0.8:1 at 55°C after 48 h of reaction time. There was negligible loss in lipase activity even after repeated use for seven cycles.

**Conclusion:**

To the best of our knowledge this is the first report on biodiesel synthesis using immobilized *E. aerogenes *lipase.

## Background

Biodiesel is a renewable fuel that can be synthesized from edible, non-edible and waste oils. Due to diminishing petroleum reserves, vegetable oils have attracted attention as a potential renewable source for the production of alternatives to petroleum-based diesel fuel. A number of processes have been developed for biodiesel production involving chemical or enzyme catalysis or supercritical alcohol treatment [1-4]. Enzymatic transesterification of triglycerides is a good alternative to chemical processes due to its eco-friendly, selective nature and low temperature requirements [5-9].

Many starting materials such as soybean oil [10,11], sunflower oil [12,13], cotton seed oil [14], rapeseed oil [15], palm oil [16,17] and restaurant kitchen wastes [18] have been evaluated for preparation of biodiesel by the enzymatic route. In many countries, like India, where edible oils are not in surplus supply, there is a need to search for alternative starting materials, such as from non-edible oils. Oil of *Jatropha curcas *(Euphorbiaceae), a non-edible oil, has been chosen for the present investigation. The seeds and oil are toxic due to the presence of toxic phorbol esters. The oil content of Jatropha seed ranges from 30 to 50% by weight, whereas in kernel the oil content ranges from 45 to 60%. The fatty acid composition of Jatropha oil consists of oleic acid 43.1%, linoleic acid 34.3%, stearic acid 6.9%, palmitic acid 4.2% and other acids 1.4%. *Jatropha curcas *is a low-growing tree, generally planted as a hedge for protecting crops from animals. It can be grown on barren land under harsh conditions and can be cultivated as a part of the strategy for reclaiming degraded lands [19]. Keeping all this in view, the Indian Government has announced a 'National Mission on Biodiesel' for Jatropha plantations in wasteland regions that is to be implemented on an area of 400,000 ha over the next five years [20].

There are many reports on biodiesel production using enzyme catalysis by free or immobilized lipases [7,8,10,15,18,21-23]. Immobilized lipase in particular is suitable for continuous biodiesel production because of the ease of its recovery from the reaction mixture. There are two major limitations of lipase-catalyzed biodiesel synthesis. One is higher cost (which can be reduced up to a certain extent by immobilization) and another is its inactivation by methanol and glycerol. It has been reported that as methanol is insoluble in vegetable oils, it inhibits the immobilized lipases and thereby decreases the catalytic activity of the transesterification reaction. Further, the hydrophilic by-product glycerol is also insoluble in the oil, so it is easily adsorbed onto the surface of the immobilized lipase leading to a negative effect on lipase activity and operational stability [24]. Use of several solvents such as n-hexane and petroleum ether in the reaction medium has been reported [25] but the problem persisted since the inhibition of lipases still occurred due to poor solubility of methanol and glycerol in the hydrophobic solvents [26]. There are some reports on enhanced biodiesel synthesis in presence of t-butanol as a solvent [27-29]. As both methanol and glycerol are soluble in t-butanol, the inhibitory effect of methanol and glycerol on lipase activity is reduced. Moreover, t-butanol is not a substrate for the lipases because it does not act on tertiary alcohols.

Biodiesel synthesis from Jatropha oil has been reported by *Chromobacterium viscosum *and *Pseudomonas cepacia *lipases. In both the cases the ethanolysis of Jatropha oil for biodiesel synthesis has been carried out [20-30]. In the present investigation, methanolysis of Jatropha oil was performed using immobilized lipase from *Enterobacter aerogenes *in the presence of t-butanol as solvent.

## Materials and methods

### Chemicals

Jatropha oil (HPLC grade) was kindly gifted by Professor P Das. Methanol was purchased from Qualigens. Methyl oleate was procured from Sigma. All other solvents and reagents were of AR grade and were obtained from Merck.

### Lipase production

All experiments were carried out using lipase from *E. aerogenes*. The extracellular lipase production from *E. aerogenes *was carried out in 250 ml Erlenmeyer flasks each containing 50 ml of a medium composed of peptone (0.5%), yeast extract (0.3%), NaCl (0.25%), MgSO4 (0.05%) and coconut oil (3.0%) at pH 7.0. Medium was sterilized and inoculated with 3.5 ml (4 × 108 cells/ml) of inoculum followed by incubation for 60 h at 34°C with shaking at 200 rpm. At the end of the incubation period, supernatant from the fermentation media was collected by centrifugation at 6987 g for 10 min. Supernatant was treated with acetone (1:4 v/v) for 1 h at 4°C followed by centrifugation at 6987 g for 10 min. The precipitate was dissolved in 50 mM phosphate buffer (pH 5.0) and lyophilized for use as a crude lipase preparation for subsequent immobilization.

### Enzyme assay

The lipase assay was performed spectrophotometrically using p-nitro phenyl palmitate as substrate [31]. p-nitro phenol was liberated from p-nitro phenyl palmitate by lipase mediated hydrolysis. One unit (U) of lipase was defined as the amount of enzyme that liberates one micromole of p-nitro phenol per minute under the assay conditions.

### Lipase immobilization

Lipase from *E. aerogenes *was immobilized on silica activated with ethanolamine followed by cross-linking with glutaraldehyde, as described previously [32].

### Reaction setup for transesterification reaction

Transesterification reaction was carried out at 30°C in screw-capped vials placed inside a reciprocal shaker. The initial reaction mixture consisted of oil:methanol molar ratio of 1:2, t-butanol:oil volume ratio of 0.2, immobilized *E. aerogene *lipase 20 U and 200 rpm (unless otherwise stated), along with the respective controls (samples without enzyme). All the experiments were performed in triplicate and the results were reported as the mean ± standard deviation.

### Sampling and analysis

Samples were taken from the reaction mixture at specified time intervals. The samples were centrifuged at 6987 g for 10 min at 4°C to remove the carrier containing the immobilized enzyme (thus negating the possibilities of additional reaction) followed by 100-fold dilution of the initial sample with n-hexane. The stability tests were performed in t-butanol in each cycle (up to 20 cycles) and the supernatant and residual immobilized enzyme activities were tested for enzyme leaching for more than seven cycles; no leaching of enzyme either in the supernatant or in the residual immobilized enzyme was observed.

Synthesis of fatty acid methyl ester was analyzed by injecting the diluted aliquots of the reaction mixture in a gas chromatograph (Agilent 6820). The column temperature was kept at 150°C for 0.5 min, raised to 250°C at 15°C/min and was maintained at this temperature for 6 min. The temperatures of the injector and detector were set at 245 and 350°C respectively. The % molar conversion of products was identified by comparing the peak areas of standard methyl esters at particular retention times. Quantification of the final products (fatty acid methyl esters) was done from the calibration curves of pure fatty acid methyl esters (methyl oleate, methyl linoleate, methyl stearate and methyl palmitate).

## Results and discussion

### Effect of substrate molar ratio

**Figure 1:**
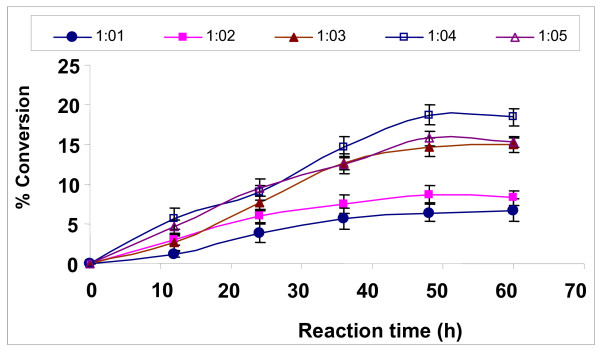
**Effect of substrate molar ratio on biodiesel synthesis**. Reaction conditions: 20 U of immobilized *E. aerogenes *lipase, t-butanol:oil volume ratio of 0.2, 30°C and 200 rpm. Data are represented as mean ± standard deviation of triplicate observation.

The optimum level of methanol concentration for the maximum synthesis of biodiesel was investigated. The transesterification reaction was carried out at 30°C, using 20 U of immobilized lipase with different molar ratios as shown in Figure 1. Alcohol in excess of the stoichiometric molar ratio of 1:3 (oil:methanol) was used to ensure higher biodiesel yield. As was expected, an increase in the number of moles of alcohol with respect to the triglycerides resulted in an increase in the production of esters. Ultimately, the formation of esters reached a maximum level with 1:4 Jatropha oil:methanol molar ratio, and a further increase in alcohol concentration resulted in a decrease in the formation of esters. The maximum biodiesel synthesis rates from rapeseed oil and cotton seed oil were reported at 1:3.6 and 1:4 oil:methanol ratio, respectively [27,29].

### Effect of agitation speed

**Figure 2:**
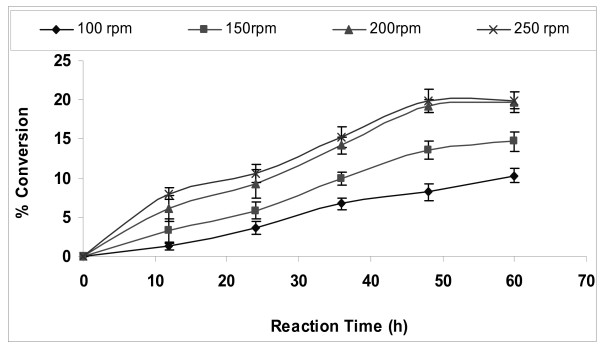
**Effect of agitation speed on biodiesel synthesis**. Reaction conditions: 1:4 oil to methanol molar ratio, 20 U of immobilized *E. aerogenes *lipase, t-butanol:oil volume ratio of 0.2 and 30°C. Data are represented as mean ± standard deviation of triplicate observation.

In case of immobilized catalyst, the reactants need to diffuse from the bulk liquid to the external surface of the particle and from there into the interior pores of the catalyst. External mass transfer limitations can be minimized by carrying out the reaction at an optimum speed of agitation [33]. The effect of speed of agitation on conversion was studied in the range of 100 to 250 rpm (Figure 2). It was found that the percentage conversion increased with speeds from 100 to 200 rpm. However, no further increase in percentage conversion was observed at 250 rpm, which may be due to shearing of the enzyme molecules. Thus, the optimum speed for transesterification reaction was found to be 200 rpm.

### Effect of t-butanol quantity

**Figure 3:**
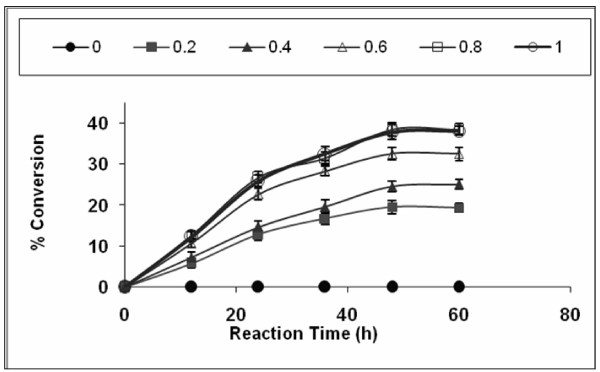
**Effect of t-butanol various t-butanol/oil volume ratio on biodiesel synthesis**. Reaction conditions: 1:4 oil to methanol molar ratio, 20 U of immobilized *E. aerogenes *lipase 30°C and 200 rpm. Data are represented as mean ± standard deviation of triplicate observation.

Biodiesel synthesis was greatly influenced by addition of t-butanol to the reaction mixture. In the absence of t-butanol there was no biodiesel synthesis, which may be due to inhibition of lipase by methanol. Generally transesterification of Jatropha oil was carried out using ethanol [20-30]. Immersion of lipases in t-butanol and other alcohols with a carbon number of more than three was claimed as a pretreatment method to increase lipase activity in synthesis of methyl esters [34,35]. Moreover, the negative effects caused by methanol and glycerol can be eliminated by the ability of t-butanol to dissolve both methanol and glycerol [23,26]. Various quantities of t-butanol were added to the reaction mixture in order to observe the effect of t-butanol concentration on the transesterification reaction. The percentage biodiesel conversion was found to increase greatly with increase in t-butanol:oil volume ratio with a maximum conversion at a t-butanol:oil volume ratio of 0.8 (Figure 3). On further increase in t-butanol concentration no increment in biodiesel synthesis was observed.

### Effect of reaction temperature

**Figure 4:**
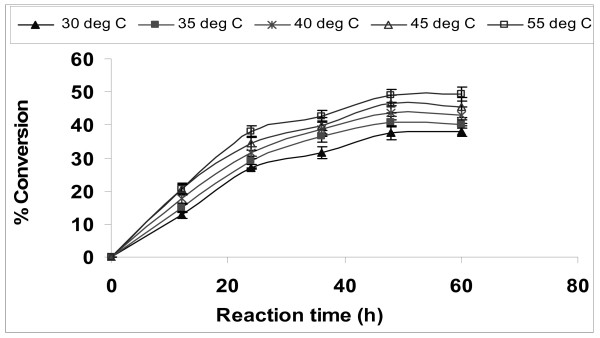
**Effect of temperature on biodiesel synthesis**. Reaction conditions: 1:4 oil to methanol molar ratio, 20 U of immobilized *E. aerogenes *lipase, t-butanol:oil volume ratio of 0.8 and 200 rpm. Data are represented as mean ± standard deviation of triplicate observation.

**Figure 5:**
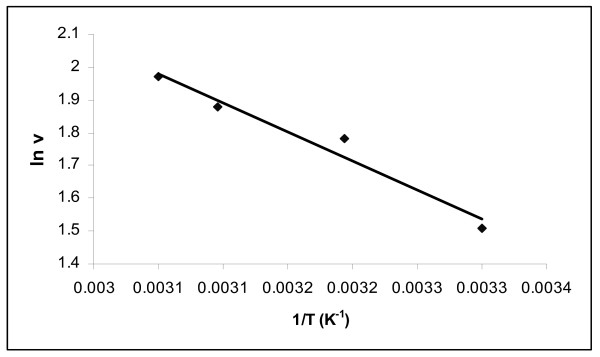
**Arrhenius plot of activation energy for biodiesel synthesis by lipase *E. aerogenes***.

An increase in temperature speeds up enzyme-mediated reactions just like any other non-enzyme catalyzed reactions. However, enzymes are proteins and are dependent on their specific structure for their activity, and may become denatured when heated beyond an optimum temperature. Experiments were performed over the temperature range of 30 to 55°C to examine the effect of temperature on the immobilized lipase for the synthesis of biodiesel at 1:4 Jatropha oil:methanol molar ratio with 20 U of immobilized lipase (Figure 4). Transesterification activity of lipase was found to continuously increase with increase in temperature, with a maximum conversion at 55°C. Royon et al also carried out the transesterification of cotton seed oil in t-butanol at 50°C [29]. The activation energy was calculated from the graph shown in Figure 5 where slope was equal to -*E*/*R*. In the present reaction, the apparent activation energy was 14.73 kJ/mol. Royon et al reported an activation energy of 19.2 kJ/mol for the transesterification of cotton seed oil [29].

### Effect of additional water content

**Figure 6:**
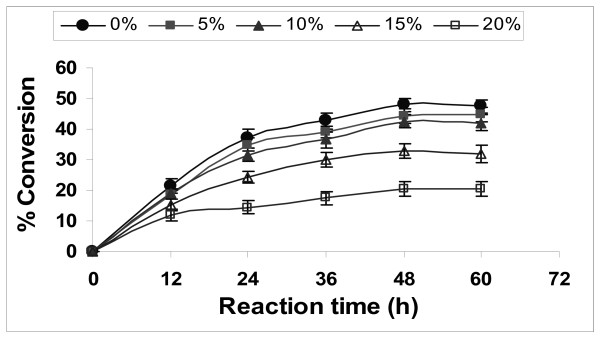
**Effect of water content on biodiesel synthesis**. Reaction conditions: 1:4 oil to methanol molar ratio, 20 U of immobilized *E. aerogenes *lipase, t-butanol:oil volume ratio of 0.8, 55°C and 200 rpm. Data are represented as mean ± standard deviation of triplicate observation.

A common thread in all studies of enzymes in organic media is that the amount of water associated with the enzyme is a key determinant of the properties (for example, activity, stability and specificity) that the enzyme exhibits. The effect of initial water concentration on the transesterification reaction was investigated through the addition of water ranging from 5 to 20% (v/v) of the total amount of reaction mixture (Figure 6). It was observed that percentage conversion was a maximum when there was no additional water in the reaction mixture. Although water is necessary for acquisition and maintenance of enzymes' catalytically active conformation in essentially anhydrous organic solvents, water is also involved in many enzyme inactivation processes [36]. As long as a minimal amount of water is associated with the enzyme, its activity in the organic media is retained. However, too much water facilitates enzyme aggregation, which leads to a decrease in enzyme activity. A similar effect was observed in the methanolysis reaction of soybean oil using lipase from *Mucor miehei *[15] and in the transesterification reaction of triolein with propanol by *Pseudomonas fluorescens *lipase [6].

### Effect of enzyme concentration

**Figure 7:**
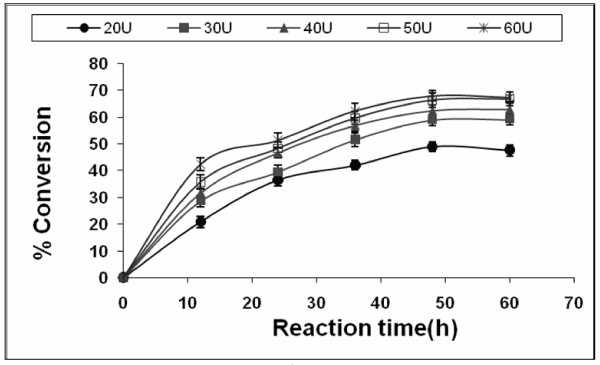
**Effect of enzyme amount on biodiesel synthesis**. Reaction conditions: 1:4 oil to methanol molar ratio, t-butanol:oil volume ratio of 0.8, 55°C and 200 rpm. Data are represented as mean ± standard deviation of triplicate observation.

The effect of enzyme concentration on the transesterification reaction was also investigated. The enzyme concentration was varied from 20 to 60 U at 1:4 Jatropha oil:methanol molar ratio and at 55°C as shown in Figure 7. The synthesis of fatty acid methyl ester initially increased with increase in the enzyme amount with maximum conversion of 68% at 50 U of immobilized *E. aerogenes *lipase, whereas upon further increase in the enzyme amount no significant increase in biodiesel synthesis was observed. A previous study has demonstrated that the yield of 1-butyl oleate increased when the amount of *Rhizopus oryzae *lipase was increased from 30 to 60 U and remained almost constant with increase in lipase amount beyond 60 U [25]. Similar results were reported for the methanolysis of rice bran oil catalysed by *Cryptococcus *spp. S-2 lipase [7]. This may be due to the fact that in the presence of a high amount of lipase, the active site cannot be exposed to the substrates and many molecules of the enzyme aggregate together. Agglomeration using immobilized lipases in a solvent-free system has been previously reported [37,38].

### Reusability of lipase

**Figure 8:**
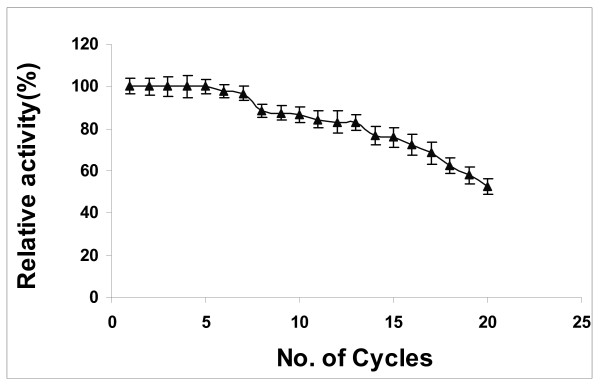
**Operational stability of *E. aerogenes *lipase for biodiesel synthesis**. Reaction conditions: 1:4 oil to methanol molar ratio, 50 U of immobilized *E. aerogenes *lipase, t-butanol:oil volume ratio of 0.8, 55°C and 200 rpm. Data are represented as mean ± standard deviation of triplicate observation.

The repeated use of immobilized enzyme may help to bring down the product cost and make the enzymatic process economically viable [39]. Operational stability or reusability is of importance in determining immobilized enzyme efficiency. However, it was observed that reaction behaviour changes when an immobilized enzyme is used repeatedly. The immobilized lipase was filtered at the end of the reaction, washed with t-butanol and again introduced with fresh reactants in order to study its operational stability in each cycle. No apparent loss in the ability of biodiesel synthesis of immobilized lipase was observed even after seven cycles of use in t-butanol system (Figure 8). This may be due to the presence of t-butanol in the reaction medium drastically reducing glycerol adsorbed on immobilized lipase, leading to enhanced lipase stability even after several uses. However, the relative activity gradually decreased to 50% after 20 cycles, which might be due to loss of enzyme during filtration and drying (since no make-up quantities of enzyme were added), prolonged interaction of the organic solvent (t-butanol) with the immobilized enzyme resulting in the denaturation of the enzyme, and the production of substantial quantities of co-product water after each cycle [40]. From this study, immobilization of lipase onto a porous support through physical adsorption has been proven to be a useful technique for improving enzyme activity through direct interaction with the lipase besides protecting it from direct inactivation by reactant and product, which is in accordance with the previous results [41].

### Fuel properties of Jatropha oil methyl esters

The fuel properties of Jatropha oil methyl ester in comparison with that of biodiesel standards has been shown in Table 1. The present results showed that the transesterification process improved the fuel properties of the oil with respect to viscosity, flash point and pour point. The comparison of these properties with diesel showed that the methyl ester has relatively closer fuel property values to that of biodiesel standard (than that of the oil). There is a substantial decrease in the viscosity compared with that of Jatropha oil, although the viscosity of the Jatropha biodiesel was slightly higher than the standard range of viscosity of biodiesel. Moreover, generally biodiesel blended with diesel is used as fuel, so no hardware modifications would be required for handling this fuel (diesel blends) in the existing engine. The flash point of Jatropha oil is lowered by transesterification, but it is still higher than that of diesel. So a small percentage addition of biodiesel with diesel increases the flash point of diesel. Hence, it is safer to store biodiesel-diesel blends as compared with diesel alone. The tested properties of methyl esters of Jatropha oil were found to be in reasonable agreement with ASTM 6751 and EN 14214.

**Table 1:** Fuel properties of methyl esters of Jatropha oil

**Property**	**Jatropha oil**	**Jatropha biodiesel**	**Biodiesel standard ASTM 6751-02**	**Biodiesel standard EN 14214**
Viscosity(mm^2^/s)	24.5	8.2	1.9 to 6.0	3.5 to 5.0
Flash point (°C)	210	8.2	>130	>120
Pour point (°C)	10	5	-15 to 10	-
Calorific value (MJ/kg)	36.2	36.5	33 to 40	-

## Conclusion

The present study shows that the efficient methanolysis of Jatropha oil is possible by lipase catalysis in presence of t-butanol as solvent. The present work is a comprehensive study on the reaction parameters influencing the enzymatic synthesis of biodiesel. Immobilized *E. aerogenes *lipase was employed to catalyze the transesterification reaction. The amount of enzyme and temperature were found to have an immense effect on biodiesel synthesis. The conversion increased with increasing temperature up to 55°C, which was near the boiling point of the reaction mixture. About 94% yield of biodiesel was obtained (of which 68% conversion was achieved with respect to methyl oleate) using 50 U of immobilized *E. aerogenes *lipase with 1:4 oil to methanol molar ratio at 55°C for 48 h. The high operational stability of immobilized lipase also indicates the efficiency of the process. From the present work, it has been demonstrated that methanolysis of Jatropha oil could be effectively carried out in this novel system with a good operational stability of the lipases. However, further research and development on additional fuel property measures, long-term run and wear analysis of biodiesel-fuelled engines is necessary.

## Competing interests

The authors declare that they have no competing interests.

## Authors' contributions

AK carried out the lipase production and transesterification study of jatropha oil. PM and VKG helped in the immobilization and fuel properties determination tasks. RB conceived of the study, and participated in its design and coordination. All authors read and approved the final manuscript.
